# One-dimensional porous nanofibers of Co_3_O_4_ on the carbon matrix from human hair with superior lithium ion storage performance

**DOI:** 10.1038/srep12382

**Published:** 2015-07-23

**Authors:** Yanli Tan, Qiuming Gao, Chunxiao Yang, Kai Yang, Weiqian Tian, Lihua Zhu

**Affiliations:** 1 Key Laboratory of Bio-inspired Smart Interfacial Science and Technology of Ministry of Education, Beijing Key Laboratory of Bio-inspired Energy Materials and Devices, School of Chemistry and Environment, Beihang University, Beijing 100191, P. R. China

## Abstract

One-dimensional (1D) hierarchical porous nanofibers of Co_3_O_4_ possessing of (220) facets on the carbon matrix from human hair (H2@Co_3_O_4_) with 20–30 nm in width and 3–5 μm in length are prepared by a facile solvothermal and calcination approach. The well crystallized small Co_3_O_4_ particles with the diameter of about 8–12 nm were closely aggregated together in the nanofibers. Electrochemical analyses show that the first discharge capacity of H2@Co_3_O_4_ electrode is 1368 mAh g^−1^ at the current density of 0.1 A g^−1^ based on the total mass of composite. A high reversible capacity of 916 mAh g ^−1^ was obtained over 100 cycles at 0.1 A g^−1^, presenting a good cycling stability. When cycled at a high current density of 1 and 2 A g^−1^, the specific capacity of 659 and 573 mAh g^−1^ could be still achieved, respectively, indicating a superior power capability.

The growing demand for developing sustainable and green-energy sources is at the top of the agenda for the whole world^1^,^2^,^3^,^4^, because of the increasing concerns on population growth and industrialization worldwide. As an electrochemical energy storage device, rechargeable lithium ion battery (LIB) is attracting widespread attention with a view to its application in popular modern electronics and hybrid electric vehicles, owing to its high energy and power density, long cyclic life, environmental friendliness and safety^5^,^6^,^7^,^8^,^9^. While there are still some challenges in the design of LIB^5^,^10^, for example high stable power density output without obvious loss of the energy density. Therefore, a variety of appealing strategies have been utilized to fabricate the electrode materials to meet the improvement of electrochemical properties of LIB.

Recently, mixed-valence spinel cobalt oxide (Co_3_O_4_) nanocrystals were paid considerable attention for use as anode material in LIB based on its excellent chemical and physical properties, such as high theoretical capacity (890 mAh g^−1^), environmental benignity, good chemical/thermal stability, highly reactive facets, and safety during operation^11^,^12^,^13^,^14^. However, the application of Co_3_O_4_ in practical LIBs is seriously hindered by the low rate capability and poor cycling performance, which are mainly caused by its low electrical conductivity and large volume change during the charge–discharge cycling. Thus, fabrication of high performance cobalt oxide-based electrode materials with satisfactory high charge/discharge rates and energy conversion efficiency is still a great challenge. Various approaches have been attempted to solve the above mentioned problems and can be mainly divided into two categories. One of the most commonly used methods is to design hybrid nanocomposites. Until now, many kinds of carbon and Co_3_O_4_ composites have been applied in LIBs, *e.g.*, graphene@Co_3_O_4_^13^,^15^,^16^,^17^, carbon nanotube (CNT)@Co_3_O_4_^18^,^19^,^20^, carbon aeroge (CA)@Co_3_O_4_^21^, onion-like carbon matrix@Co_3_O_4_^22^, and monosaccharide-derived carbon@Co_3_O_4_^23^. All of them exhibit significantly improved performances as anodes compared to pure Co_3_O_4_, because the highly conductive carbonaceous matrix can increase the electrical conductivity, prevent the aggregation of active materials and cushion the drastic volume changes. The other strategy is to prepare nanometer-sized Co_3_O_4_ with designed textures and morphologies, for example, nanowires^24^, nanosheets^11^,^25^, peapod-like^10^, nanotubes^26^, octahedral cages^27^, star-like^28^, plate-like^29^, microspheres^30^, nanorods^31^, nanobelts^32^ and particles^19^, which are believed to facilitate the electrolyte ion trapping and access to the designed nanometer-sized structures. In particular, one-dimensional (1D) hierarchical nanostructures have aroused much more interest in a variety of fields due to their rich accessible electro-active sites, fast Li^+^ ion diffusion and potential synergetic properties or multi-functionalities^33^,^34^,^35^. Notably, the research revealed that crystal facet structure is important for lithium ion transport and distinctly affects the electrochemical properties of electrode materials^14^,^19^,^26^,^36^,^37^. Thus, the 1D hierarchical Co_3_O_4_ nanocrystal materials possessing of the exposed high energy facets will be highly pursued, since their electrochemical performance is believed to be greatly improved. Electrode materials with such ideal architecture can not only provide more active unites and multiple large contact areas, but also allow fast Li^+^ ions transport between the electrolyte and electrode.

It is well known that the human hair is an easily obtained natural 1D polymer, which is mainly made up of entirely keratinized horny cells with the composition of ~51% carbon, 17% nitrogen, 21% oxygen, and so on^38^. In this paper, we report a facile approach of large-area growth for 1D hierarchical porous nanofibers of Co_3_O_4_ with (220) facets on the carbon matrix from human hair (denominated as H2@Co_3_O_4_) by solvothermal treatment of the mixture of hair-Co(CH_3_COO)_2_·4H_2_O-urea-ethylene glycol-H_2_O following with high-temperature calcination. The hair may serve as both the carbon precursor and the morphology guiding agent or “template” of the H2@Co_3_O_4_ composite nanofibers. The H2@Co_3_O_4_ nanofibers exhibit an excellent electrochemical energy stroage property as the LIB anode active material.

## Results

**Figure 1:**
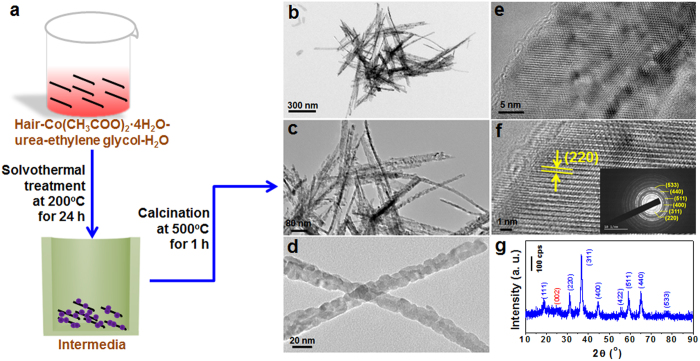
Formation process, morphology structure and phase analysis. **a**, A schematic of the synthesis steps for the H2@Co_3_O_4_ composite, which inlustrates the growth process of the sample. **b–d**, TEM images of the H2@Co_3_O_4_ composite, many 1D isolated nanobelts with 20–30 nm in width and 3–5 μm in length could be found. The small Co_3_O_4_ particles with the diameter of about 8–12 nm were closely aggregated together. **e–f**, HRTEM images of the H2@Co_3_O_4_ composite with the corresponding SAED patterns (inset of f), which show well crystallized nanostructure. And **g**, XRD patterns of the H2@Co_3_O_4_ composite.

A schematic of the growth process, morphology structures and phase analyses by the transmission electron microscopy (TEM), high resolution TEM (HRTEM), selective area electron diffraction (SAED) patterns and X-ray diffraction (XRD) patterns of the H2@Co_3_O_4_ composite are given in Fig. 1. First, the cleaned hairs were fully immersed in deionized water. Then, Co^2+^ ions were introduced into the above solution with a thorough mixing process, which enables a full adsorption of Co^2+^ ions on the surface of hair substrate through charge attraction between the positively charged cobalt ions and the negtive charged functional groups of proteins in the hair. And then, a certain amount of urea was added into the mixture, resulting in the formation of cobalt oxide precursor. After solvothermal treatment and calcination, the composite of Co_3_O_4_ on the carbon matrix from human hair (named as H@Co_3_O_4_) was obtained (Fig. 1a).

The morphologies of the as-prepared H@Co_3_O_4_ composite could be controlled by modifying the calcination time of intermediate obtained from the solvothermal treatment. Three typical kinds of H@Co_3_O_4_ composites H1@Co_3_O_4_, H2@Co_3_O_4_ and H3@Co_3_O_4_ have been investigated which correspond to the different calcination time of the precursors (see Experimental Section for detail). As shown in Fig. 1b–d and S1-2, the H1@Co_3_O_4_, H2@Co_3_O_4_ and H3@Co_3_O_4_ composites present different morphologies with varied contents of carbon from 34.76, 10.78 down to 6.15 wt.% based on the thermogravimetric (TG) measurements (Fig. S4) via increasing calcination time from 0.5, 1 to 2 h at the temperature of 500 °C in air. The H1@Co_3_O_4_ composites were thin flake-like aggregates with several nanometers in thickness, 100–300 nm in width and 1–3 μm in length (Fig. S1). The draw ratio of about 10–50 is low due to the loosely aggregated Co_3_O_4_ particles with the diameter of about 5–10 nm anchored on the carbon matrix which is easily broken under the preparation condition. Many 1D isolated nanobelts with 20–30 nm in width and 3–5 μm in length could be found for the H2@Co_3_O_4_ composites (Fig. 1b,c). The draw ratio is about 100–500, which is about ten times of that of H1@Co_3_O_4_ composites. The small Co_3_O_4_ particles with the diameter of about 8–12 nm were closely aggregated together, indicating that there may be a good conductivity for the H2@Co_3_O_4_ composite material. When further prolonging the calcination time, there were hardly isolated nanobelts but the large bundles of H3@Co_3_O_4_ composite nanobelts could be clearly observed (Fig. S2), where the Co_3_O_4_ particles became a little larger than that of H1@Co_3_O_4_ and H2@Co_3_O_4_ composites. Without adding hair in the reaction system, the as-synthesized pure Co_3_O_4_ nanoparticles could be obtained, and the particles have an average diameter of about tens of nanometers (Fig. S3). Obviously, the diameter of the Co_3_O_4_ nanoparticles of H@Co_3_O_4_ composites is much smaller than that of pure Co_3_O_4_ material. Therefore, the addition of carbon substrate from hair fiber may reduce the particle size of the Co_3_O_4_ nanoparticles and change the aggregating degree of the particles so as to control the morphology of samples. Thus, the 1D isolated H2@Co_3_O_4_ composite nanobelts are expected to show markedly excellent electrochemical properties when used as the active material of LIB anode.

Some pores or vacancy defects may be found in the H2@Co_3_O_4_ nanobelts (Fig. 1d,e), which could be favorable for providing ideal charge pathway for transports of the electrons/lithium ions when used as the electrode material. TEM images in Fig. 1e and f show that H2@Co_3_O_4_ composite nanobelts possessed well crystallized nanostructure. The clear lattice spacing of 0.285 nm fringes agreeing well with the (220) lattice spacing of face-centered cubic (*fcc*) Co_3_O_4_^14^ could be observed from Fig. 1f. The typical SAED patterns taken on the H2@Co_3_O_4_ composites (insert of Fig. 1f) can be indexed as (220), (311), (400), (511), (440) and (533) planes of the *fcc* Co_3_O_4_. The overall crystal structure and phase purity of the H2@Co_3_O_4_ composite were further identified by XRD patterns (Fig. 1g). The (111), (220), (311), (400), (422), (511), (440), and (533) peaks in the XRD patterns match well with that of the *fcc* Co_3_O_4_ (JCPDS No. 42-1467)^39^, which is consistent with the HRTEM and SAED results. Moreover, the small broad diffraction peak appeared at 2θ around 25° indexed into the (002) reflection of the graphitic-type lattice (hexagonal, space group *P*6_3_/*mmc* (No. 194), and JCPDS card No. 65-6212) represents well-developed graphitization of the carbon matrix from the hair fibers, which may be helpful for the electron transmission along the aroma carbon layers, and the low intensity is owing to the small amount of carbon (10.78 wt.%) in the composite.

**Figure 2:**
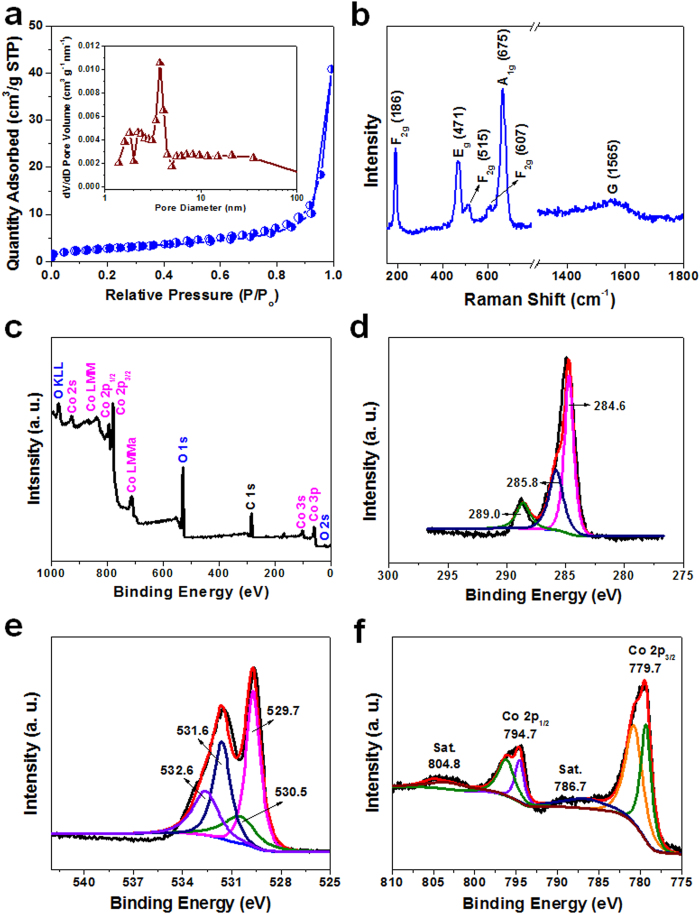
BET, Raman patterns and XPS tests. **a**, Nitrogen adsorption-desorption isotherms of the H2@Co_3_O_4_ composite. Typical IV curves were obseved, indicating the mesoporous structure. The inset is pore size distribution, showing broad pore size distribution with the average pore size of 3.73 nm. **b**, Raman spectra of the H2@Co_3_O_4_. **c**, XPS survey spectrum of the H2@Co_3_O_4_, which indicates the existence of carbon, oxygen and cobalt elements. **d**, The high-resolution spectrum of the C 1s region, where the peak at 284.6, 285.8 and 289.0 eV is corresponding to nonoxygenated carbon atoms (C-C/C = C), carbon atoms in hydroxyl groups (C-OH/C-OCo) and carbon in carboxyl groups (HO-C = O), respectively. **e**, The high-resolution spectrum of the O 1s region, where the O 1s core level spectrum is broad and four Gaussians peaks were resolved. And **f**, The high-resolution XPS spectrum of the Co 2p, which shows two major peak with binding energy at 779.7 and 794.7 eV, corresponding to the Co 2p_3/2_ and Co 2p_1/2_ peak, respectively.

It is generally accepted that the hierarchical porosity of the material has a great influence on its performance in energy storage. The adsorption-desorption isotherms of nitrogen at 77 K were obtained to measure the specific surface area and pore size distribution (Fig. 2a) using the multiple-point Brunauer-Emmett-Teller (BET) method. The isotherm of the H2@Co_3_O_4_ composite is of typical type-IV with a desorption hysteresis at a pressure range of 0.7–1.0 P/P_0_, according to the International Union of Pure and Applied Chemistry (IUPAC) classification, which suggests the presence of mesopores in the sample^40^. Moreover, when the relative pressure is close to 1, the amount of the adsorbed N_2_ rapidly increases, indicating that macropores exist in the H2@Co_3_O_4_ composite. The nitrogen adsorption-desorption isotherms and pore size distribution of the H1@Co_3_O_4_, H3@Co_3_O_4_ and pure Co_3_O_4_ are shown in Figure S5 with the corresponding textural parameters listed in Table S1. The BET specific surface area of samples decreases with the increasing Co_3_O_4_ contents. However, the average pore diameter of the sample displays a slightly decreasing trend when the Co_3_O_4_ content increases due to the blocking of some meso- and/or macropores. The pore size distribution curve of H2@Co_3_O_4_ composite determined by the Barrette-Joynere-Halenda (BJH) method (insert in Fig. 2a) showed that the pore-size distribution is broad with the average pore size of around 3.73 nm. The hierarchical pore structure of the H2@Co_3_O_4_ composite may be helpful for its electrochemical performance, since the electrolyte can penetrate more sufficiently into the pores, thus leading to a higher electrolyte/electrode contact area and more facile intercalation for Li^+^ ions in the electrolyte within the pores^41^. The BET specific surface area of the hierarchical H2@Co_3_O_4_ composite is calculated to be 42.33 m^2^ g^−1^. It is worth mentioning that the high BET specific surface area could be attributed to the porosities of the Co_3_O_4_ and carbon as well as to the formation of secondary pores between the Co_3_O_4_ and the carbon substrate. Such a pore structure can provide not only fast ionic transport channels but also sufficient buffer space for the volume expansion of Co_3_O_4_^42^. Particularly at high current density, the high surface area and typical hierarchical pore structure may shorten the Li^+^ ion diffusion path and reduce inner stress during Li^+^ ion insertion/desertion processes in the LIB system, and further leads to large capacity, high rate performance and good cycling stability.

The H2@Co_3_O_4_ composite structure was further characterized by Raman spectroscopy. Three characteristic peaks (Fig. 2b) could be observed at 186, 515 and 607 cm^−1^, which are corresponding to the F_2g_ mode of the crystalline Co_3_O_4_. The peak at 471 and 675 cm^−1^ can be attributed to the E_g_ and A_1g_ mode of Co_3_O_4_, respectively^43^. The phonon symmetries of the Raman peaks are caused by the lattice vibrations of the spinal structure, in which Co^2+^ and Co^3+^ cations are situated at tetrahedral and octahedral sites in the cubic lattice^44^. In addition, the G-band (∼1565 cm^−1^) corresponding to the sp^2^-hybridy mode of the ordered graphitic carbon was observed for the sample^45^. There is no obvious disordered carbon D-band at ∼1350 cm^−1^, indicating that the carbon substrate is lack of defects.

The surface information on the H2@Co_3_O_4_ sample was obtained by X-ray photoelectron spectroscopy (XPS). The binding energies obtained in the XPS spectra were calibrated using the C1s photoelectron peak at 284.8 eV as the reference. The sharp peak at 284.8, 531.1 and 780.7 eV (Fig. 2c) corresponds to the characteristic peak of C 1s, O 1s and Co 2p, respectively, indicating the existence of carbon, oxygen and cobalt elements. Figure 2d shows the high-resolution spectrum of the C 1s region. The peak at 284.6, 285.8 and 289.0 eV could be observed, corresponding to nonoxygenated carbon atoms (C-C/C = C), carbon atoms in hydroxyl groups (C-OH/C-OCo) and carbon in carboxyl groups (HO-C = O), respectively^46^. Figure 2e presents the high-resolution spectrum of O 1s. It can be seen that the O 1s core level spectrum is broad and four Gaussians peaks were resolved. The peak at the lower energy of 529.7 eV is associated with the lattice oxygen in the spinel Co_3_O_4_^47^. The other three peak at the higher energy of 530.5, 531.6 and 532.6 eV is associated with the oxygen in cobalt monoxide hydroxide ions, oxygen of the hydroxide ions and with the water adsorbed onto the surface of the Co_3_O_4_ nanoparticles, respectively^48^. The two forms of cobalt oxide, *i.e.*, CoO and Co_3_O_4_, can be identified by different intensities of the shakeup satellites between the main peaks Co 2p_3/2_ and Co 2p_1/2_. Figure 2f exhibits the high resolution XPS spectrum of Co 2p, which shows two major peak with binding energy at 779.7 and 794.7 eV, corresponding to the Co 2p_3/2_ and Co 2p_1/2_ peak, respectively. The gap between the peaks is about 15 eV, which is a typical characteristic of the standard Co_3_O_4_ spectra^41^. In addition, the shake-up satellite peaks are at 804.8 and 786.7 eV, confirming the existence of Co^2+^ in the sample^32^. This result indicates that the composite is composed of Co_3_O_4_ and the oxygen bridges between Co_3_O_4_ and carbon in the H2@Co_3_O_4_ composite.

**Figure 3:**
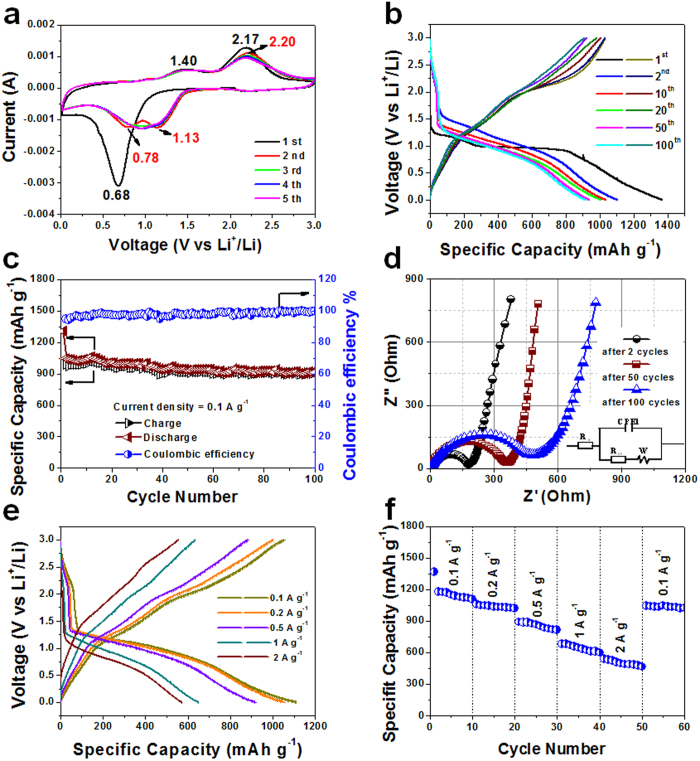
Electrochemical characterization of the H2@Co_3_O_4_ composite as anode for LIB applications. **a**, Representative CV curves at a scan rate of 0.2 mV s^−1^. **b,** Galvanostatic charge/discharge profiles for the 1^st^, 2^nd^, 10^th^, 20^th^, 50^th^ and 100^th^ cycles at 0.1 A g^−1^. **c**, Plots of charge–discharge capacities versus cycle number and Coulomb efficiency at a current density of 0.1 A g^−1^ between 0.01 and 3.0 V. **d**, EIS curves after 2, 50 and 100 cycles with the inset of the simulation model of the equivalent circuit. **e**, Charge–discharge curves at different current rates. And **f**, Rate performance at various current densities from 0.1 to 2 A g^−1^ in the voltage range of 0.01–3.0 V.

The unique structure motivates the H2@Co_3_O_4_ composite electrode with excellent lithium storage property, which was evaluated by using various electrochemical tests. Cyclic voltammetry (CV) was first conducted to investigate the electrochemical reaction process at a scan rate of 0.2 mV s^−1^ within the voltage window of 0.01–3.00 V (Fig. 3a). As to the first cathodic sweep, an irreversible peak appearing at 0.68 V was observed, which is attributed to the electrochemical reduction (lithiation) reaction of Co_3_O_4_ with Li^+^ and the formation of solid electrolyte interphase (SEI) films^49^. For the anodic sweep, the oxidation peak at 1.40 and 2.17 V was recorded, corresponding to the decomposition of SEI film and the oxidation of the Co to Co_3_O_4_, respectively^27^. Compared to the discharge-charge voltage plateaus, the cathodic peak negatively shifted and the anodic peak positively shifted due to the polarization of the electrode in the first cycle^50^. The lithium storage mechanism of the electrode can be described by the electrochemical reaction of Li with Co_3_O_4_: Co_3_O_4_ + 8Li = 4Li_2_O + 3Co. During the subsequent cycles, a decrease of the peak intensity and a shift of the potential in the positive direction were revealed compared to that of the first cycle, which indicate the occurrence of some irreversible processes in the electrode material in the initial cycle. From the second cycle, the CV curve of the sample showed two cathodic peak at 0.78 and 1.13 V, and the corresponding anodic peak was at 1.40 and 2.20 V, respectively. The pair of cathodic and anodic peaks possibly originated from the redox reaction of Co^3+^/^2+^/Co^0^ 51. Specifically, Co_3_O_4_ has a normal spinel structure with Co^2+^ and Co^3+^ ions in a cubic close packed lattice of oxide anions, so the redox reaction of Co^3+^/^2+^/Co^0^ is a complex multistep reaction behavior during the discharge processes^52^. Moreover, the two oxidation peaks hardly exhibited change in the subsequent cycles, which indicate a good reversibility and reproducibility of the electrochemical reaction for lithium ion storage. The CV behavior indicates that the overall capacity of H2@Co_3_O_4_ composite arises mainly from the properties of the metal oxide, which further shows that the excellent reversibility and stability of the electrode material have been gradually built after the initial cycle.

Typical charge/discharge curves of the H2@Co_3_O_4_ composite electrode were examined for the 1^st^, 2^nd^, 10^th^, 20^th^, 50^th^ and 100^th^ cycles based on the standard Co_3_O_4_/Li half-battery configuration at 0.1 A g^−1^ (Fig. 3b). In the first discharge curve, a long voltage plateau was shown at about 1.00 V vs Li^+^/Li and the voltage dropped gradually until the end of the discharge. The following sloping region may be relative with the reversible Li-driven decomposition of Co_3_O_4_ as well as formation of the SEI film. The voltage slope should be associated with the irreversible reactions to form the SEI film and possibly interfacial lithium storage, which can lead to an extra reversible capacity^53^,^54^. Therefore, the actual reversible capacity of Co_3_O_4_ are usually larger than the theoretical value (890 mAh g^−1^)^12^. In the following cycles, the voltage capacity curves were highly consistent indicating superior cycle stability of the H2@Co_3_O_4_ nanofibers during the lithiation-delithiation processes.

For comparison, we also measured the performance of H1@Co_3_O_4_ (Fig. S6a), H3@Co_3_O_4_ (Fig. S6b) and pure Co_3_O_4_ (Fig. S6c) prepared by the similar procedure with different calculation time under the same electrochemical conditions. The first discharge capacities of H1@Co_3_O_4_, H2@Co_3_O_4_, H3@Co_3_O_4_ and pure Co_3_O_4_ were successively 926, 1368, 1015, and 1195 mAh g^−1^ based on the total mass of composites. From the second cycle, however, the H2@Co_3_O_4_ composite electrode presented much better electrochemical lithium storage performance than the other three electrodes. Afterward, the following discharge curves tended to be stable with a value of 916 mAh g^−1^ after 100 cycles, implying that the electrochemical reactions were proceeding into the cyclable stages. The Coulombic efficiency rapidly raised from 75.4% in the first cycle to 98.3% in the 50^th^ cycle and then remained at above 99.2% in the 100^th^ cycle. As for the H1@Co_3_O_4_ and H3@Co_3_O_4_ electrodes, the discharge capacity dropped to 566 and 650 mAh g^−1^ after 100 cycles, only remaining 61.1% and 64.0% of the initial capacity, respectively (Fig. S6a and b). Compared to that of the H1@Co_3_O_4_ and H3@Co_3_O_4_ composite electrodes, the pure Co_3_O_4_ electrode showed a larger discharge capacity in the first cycle but suffered from fast reversible capacity fading, where 573 mAh g^−1^ was observed for the 50^th^ cycle and lower capacity of 293 mAh g^−1^ was gotten for the 100^th^ cycle (Fig. S6c). This result indicates that on one hand, there is a strong synergistic effect between Co_3_O_4_ nanoparticles and carbon from the hair fibers in the H@Co_3_O_4_ composites, which becomes much more apparent with cycling and plays a key role in the excellent cyclic performance of the composite; and on the other hand, it is important to note that the content of carbon from the hair substrate influences the morphology of the electrode, in which 1D nanofibers with porous structure promote the composite electrode to exhibit optimal electrochemical performance.

The cycling performance of the H2@Co_3_O_4_ electrode at a current density of 0.1 A g^−1^ was determined (Fig. 3c). The H2@Co_3_O_4_ electrode exhibited a large initial discharge capacity of 1368 mAh g^−1^, which is mainly associated with a series of irreversible reactions during the first discharge process, such as the decomposition of the electrolyte and the formation of SEI films^55^. Even for the 100^th^ cycle, the electrode still preserved a discharge capacity of 916 mAh g^−1^ and a charge capacity of 909 mAh g^−1^, corresponding to a Coulombic efficiency of 99.2%. Notably, the reversible capacity exceeds the theoretical capacity of 890 mAh g^−1^, and a rough comparison indicates that the reversible capacity is better than those previously reported^11^,^13^,^24^,^26^,^39^. However, the theoretical capacity of 890 mAh g^−1^ is predicted by the electrochemical conversion reaction mechanism and calculated by the number of transferred electronics in the reaction^53^,^56^,^57^. The excess over the theoretical value most probably arises from interfacial lithium storage in the spaces of the hierarchical pores, which indicates substantial enhancement of Li^+^ storage capacity and stability for the H2@Co_3_O_4_ electrode. Besides, the H1@Co_3_O_4_ and H3@Co_3_O_4_ electrodes showed acceptable performance with the reversible charge-discharge capacities of 558 and 639 mAh g^−1^ after 100 cycles (Fig. S7), which is about twice of the theoretical value of common commercial graphite (372 mAh g^−1^). In contrast, the pure Co_3_O_4_ exhibited a relative poor cycle performance with the reversible discharge capacity of 293 mAh g^−1^ after 100 cycles. It is worth mentioning that the addition of the carbon substrate can promote the cycling stability of Co_3_O_4_. All three H@Co_3_O_4_ composite samples have manifested excellent cycling performance compared to the pure Co_3_O_4_ electrode. The H2@Co_3_O_4_ apparently revealed the best electrochemical performance in all the four electrodes. Moreover, it once again demonstrates that the appropriate content of the carbon substrate in the composite is crucial to lithium storage property. The high electrochemical performance of H2@Co_3_O_4_ makes it one of the best known carbon and Co_3_O_4_ composite electrodes compared with the other Co_3_O_4_–based materials from different methods (Table 1). One thing to point out is that our H2@Co_3_O_4_ exhibits better initial discharge capacity and cycling stability at 0.1 A g^−1^, but the reversible discharge capacity is still lower than that of the result over Co_3_O_4_/C sample at the high current density of 2 A g^−1^, which was synthesized by using oily and expensive surfactant as the source of carbon^40^. Our carbon matrix from human hair is a readily available waste generated in barbershops and hair salons, which is natural, abundant and low cost. The green and facile solvothermal and calcination approach for our crystallized 1D hierarchical porous H2@Co_3_O_4_ nanofibers with (220) facets has the potential to large scale production. These observations suggest that the unique H2@Co_3_O_4_ fibers architecture and the advanced preparation technique are beneficial for the improvement of Co_3_O_4_ anode materials. Therefore, the H2@Co_3_O_4_ composites have great potential for Li-ion battery applications.

To further understand the electrochemical kinetics of the H2@Co_3_O_4_ electrode, electrochemical impedance spectroscopy (EIS) measurements were performed after different cycles from 0.1 MHz to 0.01 Hz, in which Z' and Z″ is the real and imaginary part of the impedance, respectively. The measured EIS spectra were analyzed and an equivalent circuit for this cell system is shown in Fig. 3d. In the Nyquist plots, the EIS at high-frequency semicircle corresponds to the resistance of the electrolyte (R_s_). The semicircle appearing in the medium frequency range is classically associated with the charge-transfer resistance (R_ct_) occurring between active materials and liquid electrolyte. The straight line at low-frequency is attributed to the diffusion of lithium ions into electrode materials, or the so-called Warburg impedance (W)^58^. The R_s_ for the H2@Co_3_O_4_ electrode was 14 Ω after 2 cycles, 2.3 Ω for the 50^th^ cycle and 2.5 Ω at the 100^th^ cycle. This phenomenon demonstrates that the electrode material needs to be activated in the electrolyte at initial cycle, so the value of the second cycle is higher than that in following cycles. When the active materials were fully infiltrated in electrolyte, the value of R_s_ may increase with increasing cycles. The R_ct_ of the H2@Co_3_O_4_ electrode increased from 190 Ω after 2 cycles to 375 Ω after 50 cycles until to 478 Ω up to the 100^th^ cycle. In general, the Li^+^ ion conductivity and diffusivity in both liquid and solid phases decrease with long-term continuous charge-discharge since the formation of the SEI membrane on the surfaces of the electrode during the repeated lithiation/delithiation process. The suitable R_ct_ values may be related to the hierarchical porosity of H2@Co_3_O_4_ composite, allowing for the diffusion of electrolyte into the pores more easily and facilitating facial charge transfer at the nanoscale unit/electrolyte interface^41^. For comparison, the EIS plots of the H1@Co_3_O_4_, H2@Co_3_O_4_, H3@Co_3_O_4_ and pure Co_3_O_4_ electrodes are shown in Figure S8. These plots clearly show that the H2@Co_3_O_4_ electrode reveals the best electrochemical kinetics among that of the H@Co_3_O_4_ and Co_3_O_4_ electrodes because of the lower ion diffusion resistance in the 1D nanofiber porous structures. The volume inflation/shrinkage or possible exfoliation of the sample during the long charge-discharge process may reduce the embedding of Li^+^ ion leading to the decline of the specific capacitance and the increase of the impedance. But, the H2@Co_3_O_4_ electrode still exhibits superior cycle stability. Consequently, the superior pore structure may accommodate the volume change of the H2@Co_3_O_4_ composite and the close contact between Co_3_O_4_ and carbon in the H2@Co_3_O_4_ composite could restraint the exfoliation of the sample during long-term cycling processes. This result indicates that the composite structure of H2@Co_3_O_4_ is beneficial for enhancing the reaction kinetics and the cycling performance of the cells during the charge/discharge process.

Figure 3e exhibits the charge and discharge profiles at different current densities of 0.1, 0.2, 0.5, 1 and 2 A g^−1^. The discharge capacity of the H2@ Co_3_O_4_ electrode at different current rate was 1110 (0.1 A g^−1^), 1054 (0.2 A g^−1^), 916 (0.5 A g^−1^), 650 (1 A g^−1^) and 571 mAh g^−1^ (2 A g^−1^), respectively. As to the voltage for charge (oxidation) in Fig. 3e, the starting voltage increased with the rising current density from 0.1 to 2 A g^−1^, which may be due to the increment in polarization voltage at the high current density. It is noteworthy that the separations between the discharge and charge plateaus were enlarged with increasing the current density. This phenomenon may arise from the kinetic effects of the porous electrode material, rendering a higher over-potential^59^. The plateau was still distinguished even at a high current density of 2 A g^−1^ as well as it was at a low current density of 0.1 A g^11^, indicating that the porous 1D H2@Co_3_O_4_ nanofiber electrode provides superb highways for fast electron transmission and electrolyte ion transport, which may greatly increase the charge rate of the electrode for high-power applications.

To demonstrate the superiority of the unique H2@Co_3_O_4_ electrode, the rate capabilities of the electrode were examined (Fig. 3f) at various current densities from 0.1 to 2 A g^−1^ and then back to 0.1 A g^−1^. The corresponding discharge capacities were varied accordingly with the discharge rates changing from 1181, to 1060, 890, 683 and 584 mAh g^−1^. The specific capacity of the H2@Co_3_O_4_ composite could reach 584 mAh g^−1^ even at a high current of 2 A g^−1^. Notably, the result is apparently superior to recently reported Co_3_O_4_-based anode materials, such as Co_3_O_4_/graphene composite showing capacity of 450 mAh g^−1^ at 2.5 A g^−1^ 18, graphene-anchored Co_3_O_4_ nanoparticle composite delivering capacity of 480 mAh g^−1^ at 500 mA g^−1^ 13, graphene-encapsulated mesoporous Co_3_O_4_ composite microspheres possessing of capacity of 264 mAh g^−1^ at 2 A g^−1^ 30, peapod-like Co_3_O_4_/carbon nanocomposites exhibiting capacity of 400 mAh g^−1^ at 1 A g^−1^ 10, Co_3_O_4_/carbon composite nanowires demonstrating capacity of 358 mAh g^−1^ at 800 mA g^−1^ 60, and graphene-coated Co_3_O_4_ fibers showing capacity of 295 mAh g^−1^ at 1 A g^−1^ 49. Remarkably, when the current density returned to 0.1 A g^−1^, a capacity of 1043 mAh g^−1^ was recovered, indicating the electrode structure remains stable even under the high rate of cycling.

## Discussions

We have demonstrated the rational design and fabrication of the hierarchically porous carbon and Co_3_O_4_ nanocomposites through a facile solvothermal and calcination approach. The content of carbon substrate from hair fiber adjusted by modifying the calcination time of precursor, which not only may reduce the particle-size to form homogeneous nanoparticles but also change the aggregating degree of the particles so as to control the morphology and pore texture of samples. The optimized 1D hierarchical porous H2@Co_3_O_4_ nanofibers with (220) facets served as an anode for LIB applications show a large specific discharge capacity, excellent cycle stability, and high power output characteristic. The advanced preparation technique and high performance suggest that H2@Co_3_O_4_ composite materials have great potential in various energy storage technologies.

## Methods

### Materials preparation

The typically straight, middle thickness and black Asian hairs were used for all the experiments, which were collected from a healthy Chinese volunteer in Beihang University. The hair fibers were thoroughly washed with isopropanol and dried at 80 °C. The cleaned fibers were cut into fine debris (~2 mm in length). All the chemicals used in the experiments are analytical grade and were used without further purification. The precursors are synthesized under hydrothermal condition. In a typical synthesis, 2.0 mmol of Co(CH_3_COO)_2_·4H_2_O was dissolved in 40 mL of a mixture containing 3.0 mL of ethylene glycol and 37 mL of deionized water. After stirring for 15 min, 0.11 g of urea was added into the above solution. The mixture was stirred for another 30 min. Then 0.2 g of cleaned hair fibers were added into the above solution and immersed for 1 h. The obtained mixture was transferred into a 50 mL Teflon-lined stainless steel autoclave. The autoclave was sealed and maintained at 200 °C for 24 h in an electron oven. After that, the autoclave was cooled naturally to room temperature. The product was collected and washed with deionized water and ethanol for several times by centrifugation, followed by vacuum-drying at 60 °C. After calcinating the collected precursor at 500 °C in air for different time (0.5, 1 and 2 h), the porous H@Co_3_O_4_ composite was obtained, which is accordingly named as H1@Co_3_O_4_, H2@Co_3_O_4_ and H3@Co_3_O_4_, respectively. For comparison, pure Co_3_O_4_ sample was synthesized under the same synthetic condition as that of H2@Co_3_O_4_ without the hair in the preparation system.

### Characterization

TEM and HRTEM were examined on JEOL JEM-2100F at an acceleration voltage of 200 kV. Powder XRD patterns were determined on the X-ray diffractometor (X-ray 6000) with the 2θ angle region from 10° to 90° at a scan rate of 3° min^−1^. N_2_ adsorption-desorption isotherms were examined at 77 K using a Micromeritics ASAP 2020. Raman spectra were obtained on a microscopic confocal Raman spectrometer (Lab RAM HR800) under a back scattering geometry (λ = 514 nm). XPS analyses were performed using an Al Kα (150 W) monochromatic X-ray source (ESCALAB 250, Thermo Fisher Scientific, USA). TG analyses were determined at SDTQ600 (TA Instruments, USA) under an air atmosphere at a heating rate of 10 °C min^−1^ from room temperature to 800 °C. CV was performed by using CHI1040C electrochemical work station between 0.01 and 3.00 V at a scan rate of 0.2 mV s^−1^. The galvanostatic charging/discharging test was conducted by using coin cells (CR2032) at room temperature on a multi-channel battery testing system (LAND CT2001A) with a cutoff voltage of 3.00–0.01 V vs Li^+^/Li. Working electrodes were prepared by mixing 80 wt.% the Co_3_O_4_ or H@Co_3_O_4_ material, 10 wt.% acetylene black (Super-P), and 10 wt.% polyvinylidenefluoride (PVDF) binder dissolved in N-methyl-2-pyrrolidinone (NMP). 1.0 M LiPF_6_ in mixed ethylene carbonate (EC) and diethyl carbonate (DEC) (EC: DEC = 1:1 by volume) was used as the electrolyte in the system. EIS measurements were conducted for the working electrode in a frequency range of 0.1 MHz to 0.01 Hz.

## Additional Information

**How to cite this article**: Tan, Y. *et al.* One-dimensional porous nanofibers of Co3O4 on the carbon matrix from human hair with superior lithium ion storage performance. *Sci. Rep.* **5**, 12382; doi: 10.1038/srep12382 (2015).

## Supplementary Material

Supplementary Information

## Figures and Tables

**Table 1:** The specific capacity of H2@Co_3_O_4_ nanofibers compared with the reported results on the Co_3_O_4_-based materials with different morphologies.

Material	Morphology	Current density [mA g^−1^]	The first discharge/charge capacity [mAh g^−1^]	Initial Coulombic efficiency [%]	Capacity [mAh g^−1^] after (x) cycles	Ref.
Co_3_O_4_-graphene	nanosheet	143	1430/730	51.1	630 (50)	16
Co_3_O_4_/r-GO	nanowall	180	1236/707	57.2	673 (100)	15
Co_3_O_4_/C	nanoplate	100	1254/1035	82.5	1079 (50)	40
Co_3_O_4_/CNT	hollow	50	1420/977	68.8	977 (100)	20
G-Co_3_O_4_	microsphere	100	1533/1266	82.6	820 (35)	30
Co_3_O_4_@carbon	peapod-like	890	1800/1050	58.3	800 (50)	10
Co_3_O_4_/graphene	nanoparticle	58	1097/753	68.6	800 (30)	13
Co_3_O_4_-graphene	hexagonal ring	178	1029/750	72.9	748 (50)	17
H2@Co_3_O_4_	nanofiber	100	1368/1031	75.4	916 (100)	Our work
