# Supplementary material for: One-dimensional porous nanofibers of Co3O4 on the carbon matrix from human hair with superior lithium ion storage performance
Source: Sci Rep. 2015 Jul 23;5:12382. doi: 10.1038/srep12382 (PMC4511864; doi:10.1038/srep12382)
Supplement: Supplementary Information [file srep12382-s1.pdf]

Supplementary information

One-dimensional porous nanofibers of Co_3O_4 on the carbon matrix from human hair with superior lithium ion storage performance

Yanli Tan[1], Qiuming Gao[1*], Chunxiao Yang[1], Kai Yang[1], Weiqian Tian[1] & Lihua Zhu[1]

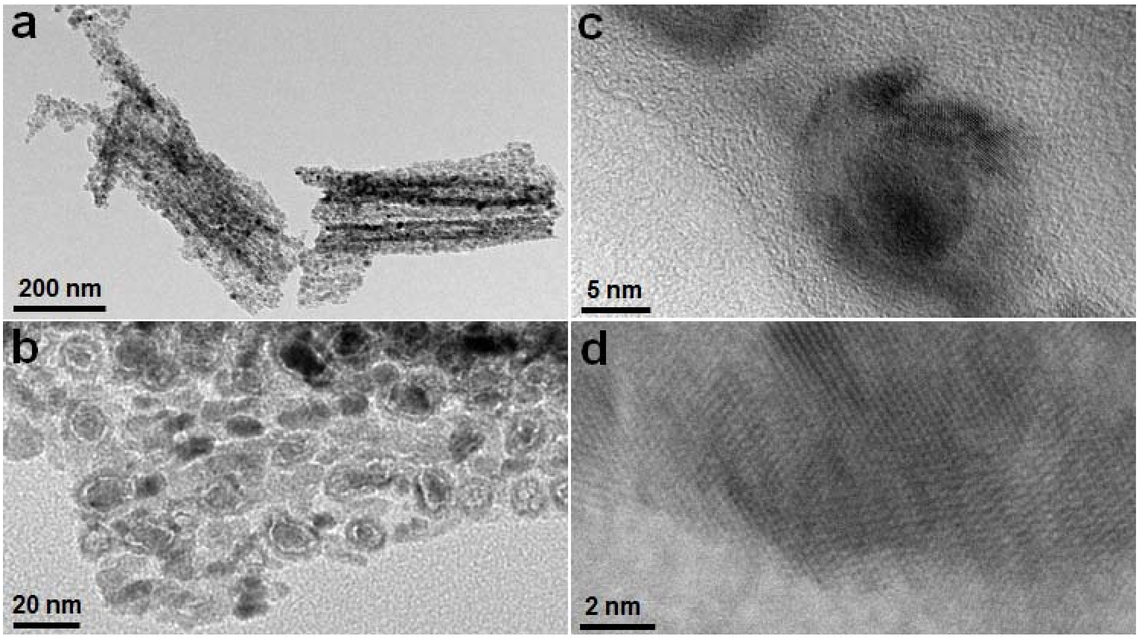

Supplementary Figure S1: (a and b) TEM and HRTEM (c and d) of H1@Co_3O_4 prepared by solvothermal treatment of the mixture of hair-Co($CH_3COO)_2$·4H_2O-urea-ethylene glycol-H_2O following with high-temperature calcination at 500℃ for 0.5 h. The H1@Co_3O_4 composites were thin flake-like aggregates with several nanometers in thickness, 100-300 nm in width and 1-3 μm in length.

---

[1]Key Laboratory of Bio-inspired Smart Interfacial Science and Technology of Ministry of Education, Beijing Key Laboratory of Bio-inspired Energy Materials and Devices, School of Chemistry and Environment, Beihang University, Beijing 100191, P. R. China. Correspondence and requests for materials should be addressed to Q.-M.G. (E-mail: qmgao@buaa.edu.cn).

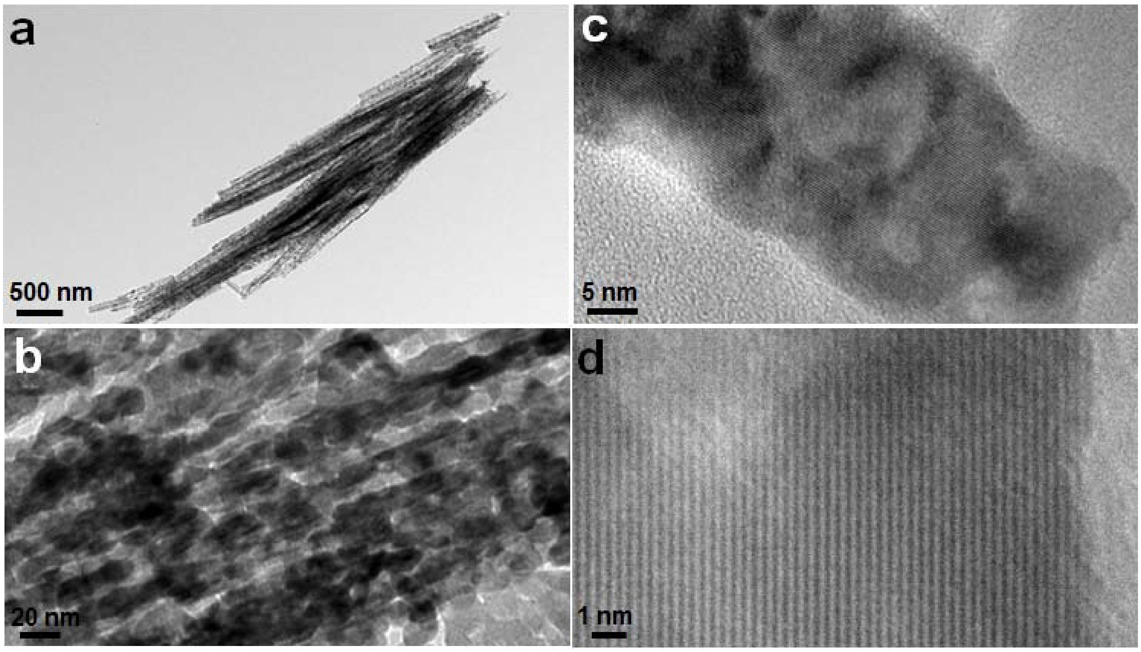

Supplementary Figure S2: (a and b) TEM and HRTEM (c and d) of H3@Co_3O_4 prepared by solvothermal treatment of the mixture of hair-$Co(CH_3COO)_2{\cdot}4H_2O$-urea-ethylene glycol-$H_2O$ following with high-temperature calcination at 500℃ for 2 h. There were hardly isolated nanobelts but the large bundles of H3@Co_3O_4 composite nanobelts could be clearly observed, where the Co_3O_4 particles became a little larger than that of other H@Co_3O_4 composites.

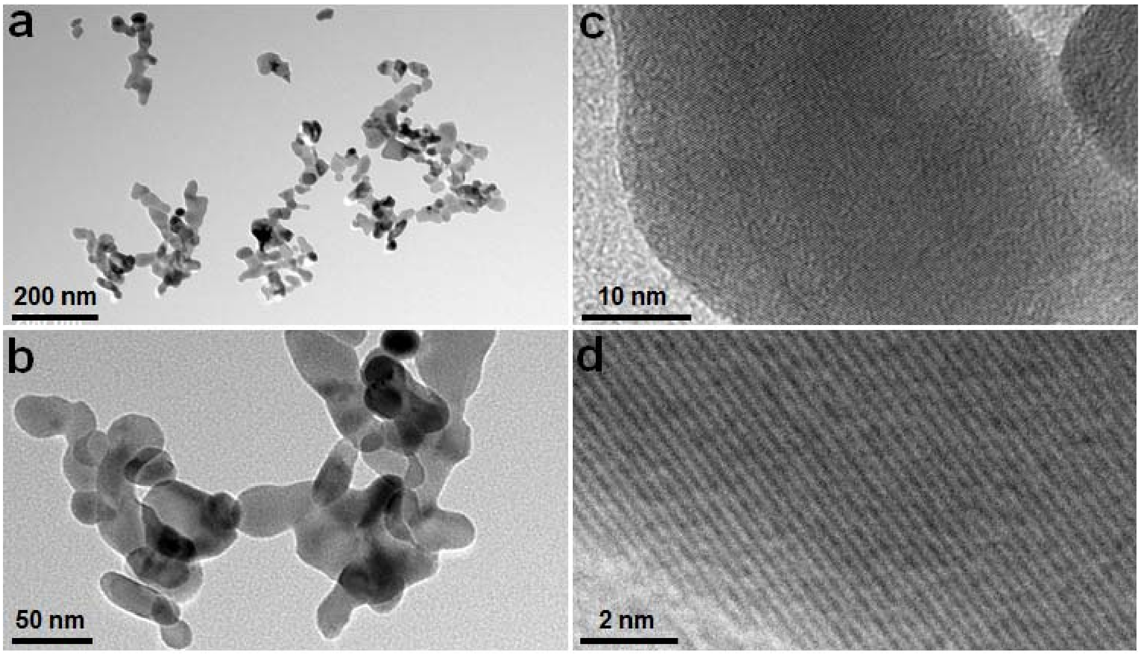

Supplementary Figure S3: (a and b) TEM and HRTEM (c and d) of pure Co_3O_4 prepared by solvothermal treatment of the mixture of $Co(CH_3COO)_2{\cdot}4H_2O$-urea-ethylene glycol-$H_2O$ following with high-temperature calcination at 500℃ for 1 h. The pure Co_3O_4 particles have an average diameter of about tens of nanometers, which is much larger than that of H@Co_3O_4 composite material.

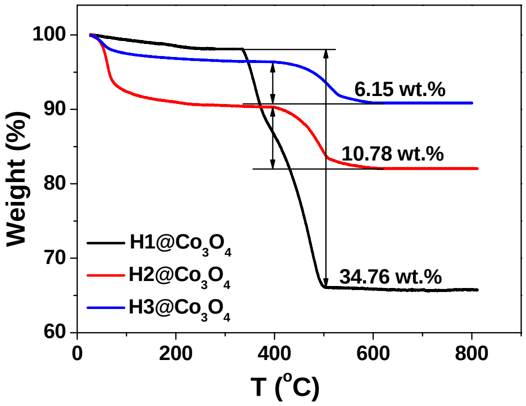

Supplementary Figure S4: TGA curves of H@Co_3O_4 composites under an air atmosphere at a heating rate of 10℃ min^{-1} from room temperature to 800℃. The H1@Co_3O_4, H2@Co_3O_4 and H3@Co_3O_4 composites present varied contents of carbon from 34.76, 10.78 down to 6.15 wt.% via increasing calcination time from 0.5, 1 to 2 h at the temperature of 500℃ in air.

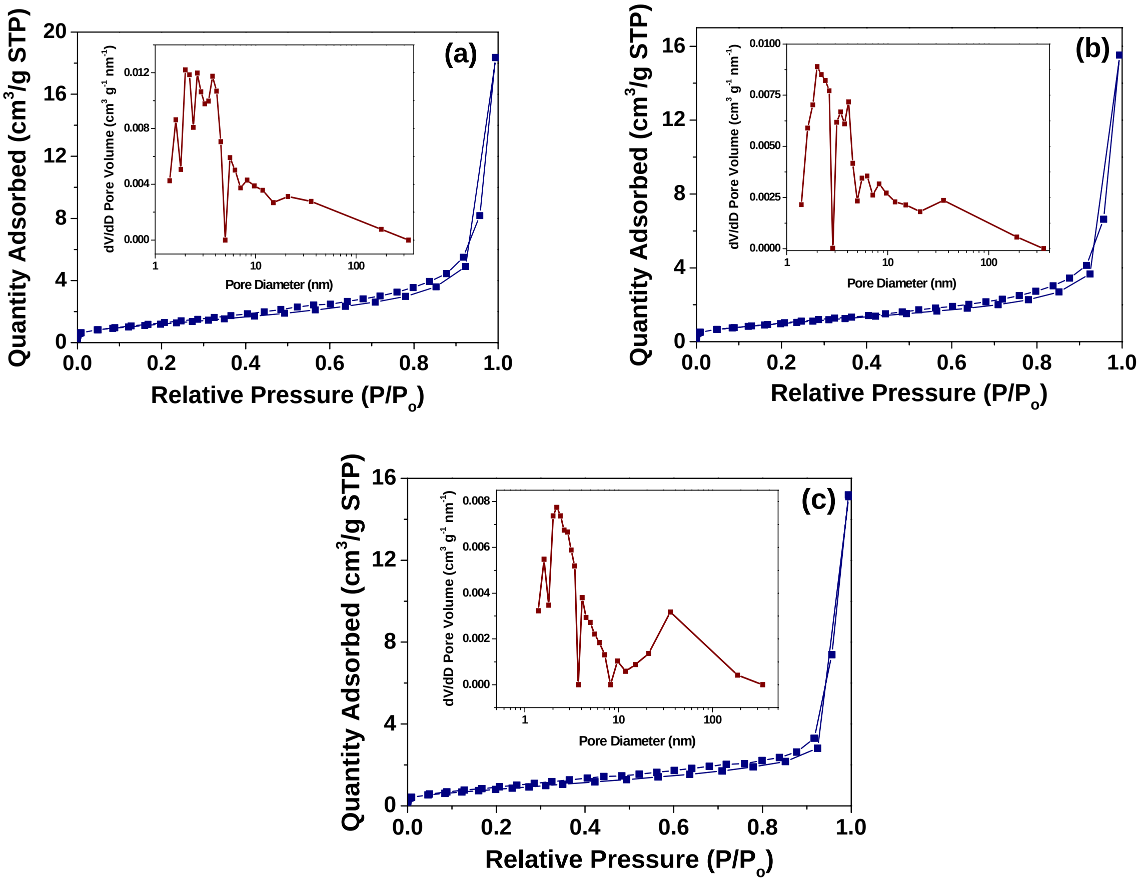

Supplementary Figure S5: Nitrogen adsorption-desorption isotherms and the inset pore size distribution curves of the electrodes with different Co_3O_4 contents for the samples of (a) H1@Co_3O_4, (b) H3@Co_3O_4 and (c) Co_3O_4. The BET specific surface area of samples decreases with the increasing Co_3O_4 contents. However, the average pore diameter of the sample displays a slightly decreasing trend when the Co_3O_4 content increases due to the blocking of some meso- and/or macropores.

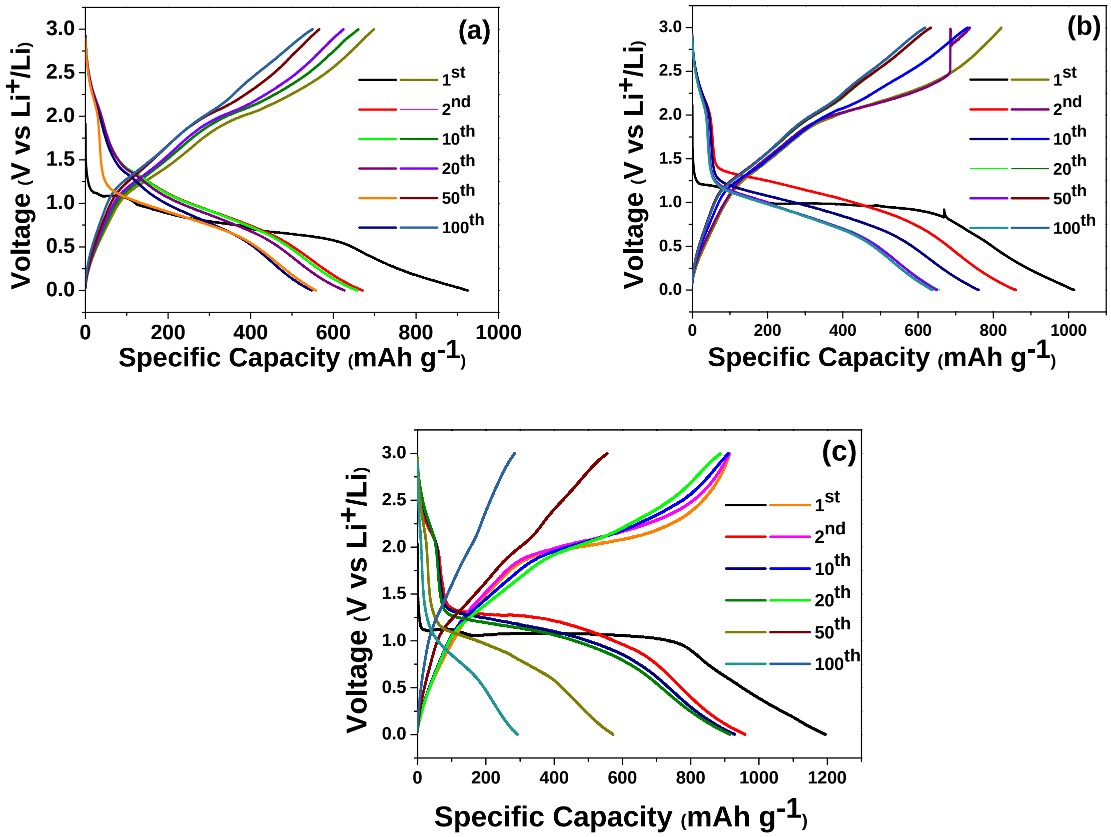

Supplementary Figure S6: Charge-discharge profiles of the electrodes with different Co_3O_4 contents for cycles at a current density of 0.1 A g^{-1}: (a) H1@Co_3O_4; (b) H3@Co_3O_4; and (c) Co_3O_4. The discharge capacity dropped to 566 and 650 mAh g^{-1} after 100 cycles, only remaining 61.1% and 64.0% of the initial capacity, respectively (a and b). Compared to that of the H1@Co_3O_4 and H3@Co_3O_4 composite electrodes, the pure Co_3O_4 electrode showed a larger discharge capacity in the first cycle but suffered from fast reversible capacity fading, where 573 mAh g^{-1} was observed for the 50th cycle and lower capacity of 293 mAh g^{-1} was gotten for the 100th cycle (c).

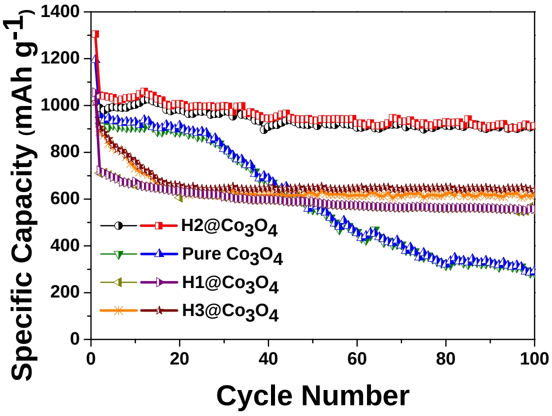

Supplementary Figure S7: Cycling performance of the electrodes with different Co_3O_4 contents. All three H@Co_3O_4 composite samples have manifested excellent cycling performance compared to the pure Co_3O_4 electrode. The H2@Co_3O_4 apparently revealed the best electrochemical performance in all the four electrodes, demonstrating that the appropriate content of the carbon substrate in the composite is crucial to lithium storage property.

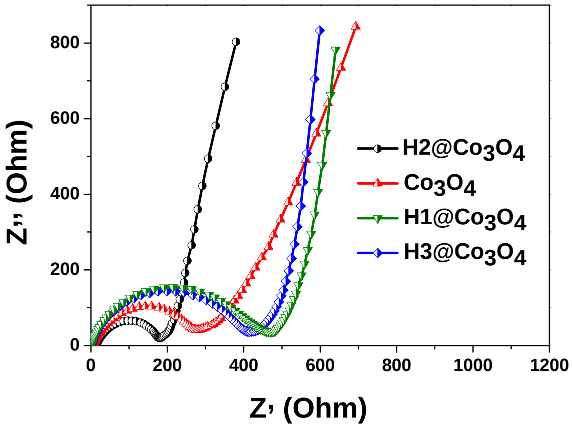

Supplementary Figure S8: The Nyquist plots of the electrodes with different Co_3O_4contents. These plots clearly show that the H2@Co_3O_4 electrode reveals the best electrochemical kinetics among that of the H@Co_3O_4 and Co_3O_4 electrodes because of the lower ion diffusion resistance in the 1D nanofiber porous structures.

Supplementary Table S1: Pore parameters for the H@Co_3O_4 and Co_3O_4 samples.

Sample	S_{BET} [m^2 g^{-1}] a)	V_t [cm^3 g^{-1}] b)	D_{BJH} [nm] c)
H1@Co_3O_4	56.11	0.364	2.000
H2@Co_3O_4	42.33	0.280	3.726
H3@Co_3O_4	42.31	0.281	2.197
Co_3O_4	28.46	0.223	1.999

a) S_{BET} represented the BET surface area; b) V_t was on behalf of the total pore volume; and c) D_{BJH} was representative of the BJH desorption average pore diameter.
